# A Promising Needle-Free Pyro-Drive Jet Injector for Augmentation of Immunity by Intradermal Injection as a Physical Adjuvant

**DOI:** 10.3390/ijms24109094

**Published:** 2023-05-22

**Authors:** Jukito Sonoda, Izuru Mizoguchi, Shinya Inoue, Aruma Watanabe, Ami Sekine, Miu Yamagishi, Satomi Miyakawa, Natsuki Yamaguchi, Eri Horio, Yasuhiro Katahira, Hideaki Hasegawa, Takashi Hasegawa, Kunihiko Yamashita, Takayuki Yoshimoto

**Affiliations:** 1 Department of Immunoregulation, Institute of Medical Science, Tokyo Medical University, 6-1-1 Shinjuku, Shinjuku-ku, Tokyo 160-8402, Japan; 2 Department of Device Application for Molecular Therapeutics, Graduate School of Medicine, Osaka University, CoMIT 0603, 2-2 Yamada-oka, Suita, Osaka 565-0871, Japan

**Keywords:** pyro-drive jet injector, needle-free injector, intradermal injection, vaccine, CTL generation, antibody production, shear stress, SARS-CoV-2, COVID-19

## Abstract

Current worldwide mRNA vaccination against SARS-CoV-2 by intramuscular injection using a needled syringe has greatly protected numerous people from COVID-19. An intramuscular injection is generally well tolerated, safer and easier to perform on a large scale, whereas the skin has the benefit of the presence of numerous immune cells, such as professional antigen-presenting dendritic cells. Therefore, intradermal injection is considered superior to intramuscular injection for the induction of protective immunity, but more proficiency is required for the injection. To improve these issues, several different types of more versatile jet injectors have been developed to deliver DNAs, proteins or drugs by high jet velocity through the skin without a needle. Among them, a new needle-free pyro-drive jet injector has a unique characteristic that utilizes gunpower as a mechanical driving force, in particular, bi-phasic pyrotechnics to provoke high jet velocity and consequently the wide dispersion of the injected DNA solution in the skin. A significant amount of evidence has revealed that it is highly effective as a vaccinating tool to induce potent protective cellular and humoral immunity against cancers and infectious diseases. This is presumably explained by the fact that shear stress generated by the high jet velocity facilitates the uptake of DNA in the cells and, consequently, its protein expression. The shear stress also possibly elicits danger signals which, together with the plasmid DNA, subsequently induces the activation of innate immunity including dendritic cell maturation, leading to the establishment of adaptive immunity. This review summarizes the recent advances in needle-free jet injectors to augment the cellular and humoral immunity by intradermal injection and the possible mechanism of action.

## 1. Introduction

The current worldwide success of vaccines against the severe acute respiratory syndrome coronavirus-2 (SARS-CoV-2), BNT162b2 [1] and mRNA-1273 [2] has attracted great interest in the prophylactic effectiveness and safety of mRNA vaccines by intramuscular injection using a needled syringe [3]. Since Edward Jenner established the concept of vaccines and developed the first vaccine against smallpox more than 200 years ago, vaccines have saved hundreds of millions of lives from infection worldwide and their safety and effectiveness have been globally proven [4]. The administration of vaccines is now one of the most powerful and successful therapies against infectious diseases caused by viruses (e.g., smallpox, polio and measles) and bacteria (e.g., tuberculosis, diphtheria and tetanus), cancers such as cervical cancer caused by human papillomavirus (HPV) infection and hepatitis caused by hepatitis B virus (HBV) infection, as well as other hematological and solid cancers [5]. Vaccination is a biological process that activates innate immunity by vaccine and consequently establishes adaptive immunity to a particular infectious disease and cancer. A vaccine typically contains a target antigen protein and adjuvant that activates innate immunity, such as ligands for toll-like receptors (TLRs) or activators of inflammasome, leading to the establishment of adaptive immunity [6].

Although the coronavirus disease 2019 (COVID-19) mRNA vaccines are saving many lives, associated adverse effects with very low frequencies following vaccination have been reported, such as acute myocardial infraction, Guillan-Barr syndrome, cerebral venous sinus thrombosis pulmonary embolism, stroke, herpes zoster reactivation and autoimmune diseases [7,8,9]. These adverse effects might be explained by the following possible mechanisms. COVID-19 mRNA is encapsulated in lipid nanoparticles (LNPs) [10], and the LNPs are probably broadly distributed in a human body and may exert a proinflammatory action [11]. Moreover, the COVID-19 mRNA encodes a transmembrane SARS-CoV-2 spike (S) protein, and the antigen and its related peptide fragments may be shed into the circulation. Consequently, the binding of the circulating S protein or related peptide fragments to the receptor angiotensin-converting enzyme 2 (ACE2) [12,13] may be involved in the vaccination-related adverse effects [14].

Due to the fact that the skin has numerous immune cells, such as professional antigen-presenting dendritic cells (DCs), the skin is the ideal site for vaccination [15]. A significant amount of evidence revealed that intradermal injection is superior to intramuscular injection in that intradermal injection elicits more potent immunity than intramuscular injection [16,17,18,19,20]. However, intradermal injection requires more proficiency than intramuscular injection and is therefore considered not suitable for mass vaccination. In addition, the injection of drugs using a needled syringe is often accompanied by pain and needle phobia, which are the main reasons for hesitation in receiving vaccination [21]. Therefore, less painful and more efficient and easy injection procedures are highly desired for vaccination and drug delivery into the skin.

To meet these desires, i.e., to more painlessly and efficiently augment the cellular and humoral immunity by intradermal injection, several delivery devices have been developed including jet injectors, electroporation, thermal ablation, iontophoresis, ultrasound and microneedles [15]. Among them, this review summarizes the recent advances in the needle-free jet injectors and the possible mode of action. To date, several versatile needle-free jet injectors have been developed, and among them this review has highlighted a new device for a needle-free pyro-drive jet injector (PJI) and introduced its unique characteristics and advantages as a tool to induce protective immunity by vaccination toward cancers and infectious diseases, such as SARS-CoV-2.

## 2. Intradermal Injection

**Figure 1:**
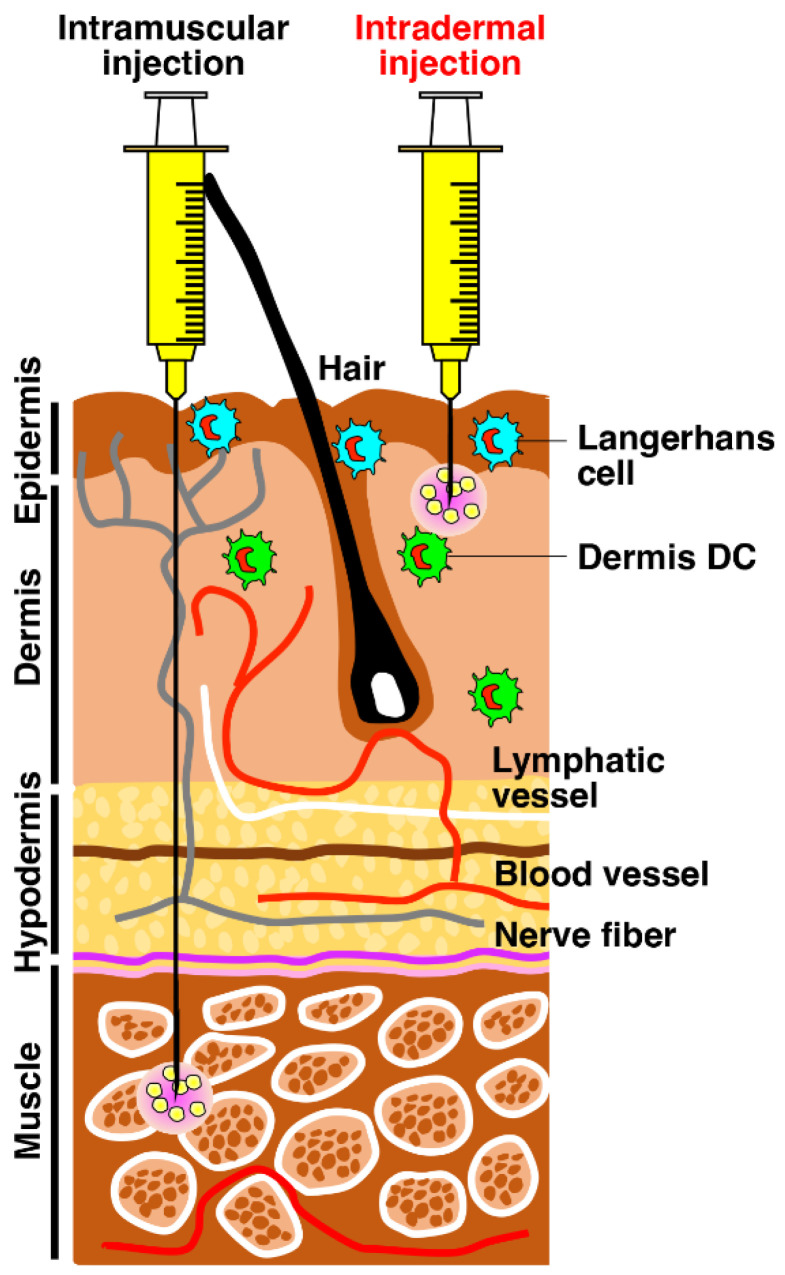
Intradermal injection is superior to intramuscular injection for induction of cellular and humoral immunity. Intramuscular injection is generally well tolerated, safer and easier to perform on a large scale. In contrast, the skin has the benefit of the presence of numerous immune cells, such as professional antigen-presenting DCs, and plays the frontline to initiate innate immunity leading to protective and pathogenic immune responses [16,17,18,19,20]. Therefore, intradermal injection is considered superior to intramuscular injection for the induction of protective immunity although more proficiency is required for the injection.

Human muscle tissue is rich in blood supply, and vaccine antigens administered into the muscle are rapidly absorbed into the blood circulation (Figure 1). In contrast, the skin is highly immunogenic and consists of several layers, such as epidermis, dermis and hypodermis, from the outside to the muscle [15]. The stratum corneum in the epidermis is composed of several layers of dead keratinocytes, humectants, lipids and proteins [22,23]. This is the frontline barrier against exposure to pathogens and foreign antigens, leading to protective immunity against pathogens and elicited sensitization to foreign antigens leading to allergic diseases [22,23]. The epidermal basal stratum is dominated by keratinocytes, which differentiate and migrate to the stratum corneum, playing a crucial role in innate immune responses via pattern recognition receptors [24] and inflammatory cytokines. The dermis is mainly composed of fibroblasts with collagen and elastin fibers for the elasticity and strength of the skin and is rich in lymphatic vessels and blood capillaries (Figure 1) [20,25]. There are many resident professional antigen-presenting DCs, such as Langerhans cells in the epidermis and dermis DCs that play indispensable roles in orchestrating and establishing adaptive immunity [26]. DCs capture antigens, process them, distribute them on their MHC classes I and II and migrate to the draining lymph node to stimulate the antigen-specific naïve T cells and consequently initiate adaptive immunity [27]. The hypodermis is composed of adipose tissue, which forms vacuoles and plays the role of a reservoir of interstitial fluid in providing the stiffness of the skin [28].

Current mRNA vaccines against SARS-CoV-2 are intramuscularly administered worldwide with great successes [3]. A significant amount of evidence has revealed that intradermal injection is superior to intramuscular injection in that intradermal injection elicits more potent protective cellular and humoral immunity than intramuscular injection [16,17,18,19,20]. This difference is considered to be due to the presence of numerous immune cells, such as professional antigen-presenting DCs in the skin. Therefore, the administration dosage is reduced by approximately 5~10 times. However, intradermal injection requires more proficiency than intramuscular injection and is therefore considered unsuitable for mass vaccination.

## 3. Needle-Free Jet Injector

The most common procedure to inject drugs is hypodermic injection with a needle injector. However, this procedure is often accompanied with pain and needle phobia, which are the main reasons for hesitation in receiving vaccination [21,29]. Moreover, the risks of cross-infection with HBV, HCV and human immunodeficiency virus (HIV) by reuse of needles and the issues of hazardous waste after injection using needled syringes were reported [30,31]. In contrast, needle-free intradermal injection has several advantages in that it is easy to operate, is dose-sparing due to intradermal efficiency, involves no possibility of cross-contamination by re-use and needle-stick injuries and is suitable to large-scale vaccination [32].

**Figure 2:**
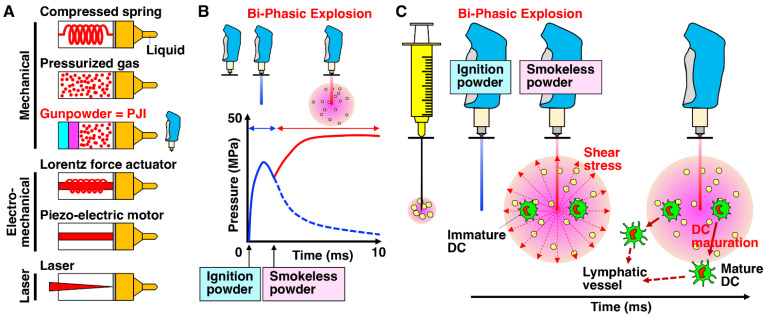
Various types of needle-free jet injectors with different driving forces. (**A**) In contrast to the typical needled syringe, various types of needle-free injectors with different driving forces have been developed. These needle-free injectors possess several advantages in that they are easy to operate, are dose-sparing, do not present the issue of cross-contamination by re-use, do not cause needle-stick injuries and are suitable for large-scale vaccination [38,39,40,41]. (**B**) Among them, a new needle-free pyro-drive jet drive injector (PJI) is a unique device that utilizes two different types of explosives, ignition and smokeless powders and can control the penetration depth and wide dispersion of delivery [42]. (**C**) The high-speed camera analysis in a polyurethane gel revealed that the liquid rapid jet creates a hole in the gel upon the initial explosion caused by the ignition powder, penetrates and then ceases the penetration [42]. Thereafter, upon the second explosion induced by the smokeless powder, the liquid jet disperses in a concentric circle around the end of the formed hole. Shear stress generated by rapid jet velocity via the PJI accelerates the uptake of plasmid DNA and, consequently, its protein expression, followed by the induction of DC maturation and migration through lymphatic vessels [43].

Adjuvant is a substance that activates innate immunity leading to the establishment of adaptive immunity, thus providing enhanced immunity to a particular pathogen. Examples for it are as follows: so-called danger signals [33,34]; pathogen-associated molecular patterns (PAMPs), such as components of bacterial cell walls; damage-associated molecular patterns (DAMPs), such as DNA, RNA and high-mobility group box 1 (HMGB1); and inflammasome activators, such as adenosine triphosphate (ATP) and uric acid. So far, only a limited number of adjuvants have been approved for use in humans due to toxicity, and the aluminum salt alum is the most widely used adjuvant worldwide [33,35]. Alum potently augments mainly helper CD4^+^ T (Th)2 immune responses and consequent humoral immune responses in part through the induction of uric acid, one of the endogenous danger signals [33,34]. Due to the safety concerns of such chemical adjuvants, safer and more efficient methods are currently required. Physical adjuvants, which do not use any chemical and biological substances as adjuvants, and therefore do not remain in the exposed tissues nor induce persistent adverse effects, have recently attracted great interest [36,37,38]. The physical adjuvants utilize physical energies to provide a kind of stress to tissues and cells to accelerate the uptake of molecules and the release of danger signals, consequently eliciting innate immune responses [38]. To date, various types of needle-free jet injectors have been developed to deliver drugs or antigens into the skin using a compressed spring and pressurized gas as an electromechanical driving force, such as the Lorentz and Piezo forces (Figure 2A) [39,40,41]. Further, laser energy has been incorporated recently as an effective driving force. In the laser-assisted vaccination, the liquid that absorbs the optical laser energy gives rise to explosively growing bubbles, which then induce a fast microfluidic jet, resulting in the penetration of the skin [38,41]. In addition, a significant amount of evidence revealed that laser light has an ability to enhance the immune responses to injected antigen, following the release of heat shock protein 70 (HSP70) as an immunologic adjuvant. Therefore, this is called a laser adjuvant [36,37]. However, most of them present difficulties in the control of jet pressure to adjust the injection volume and penetration depth, or the miniaturization of actuators.

## 4. A New Needle-Free Pyro-Drive Jet Injector

**Figure 3:**
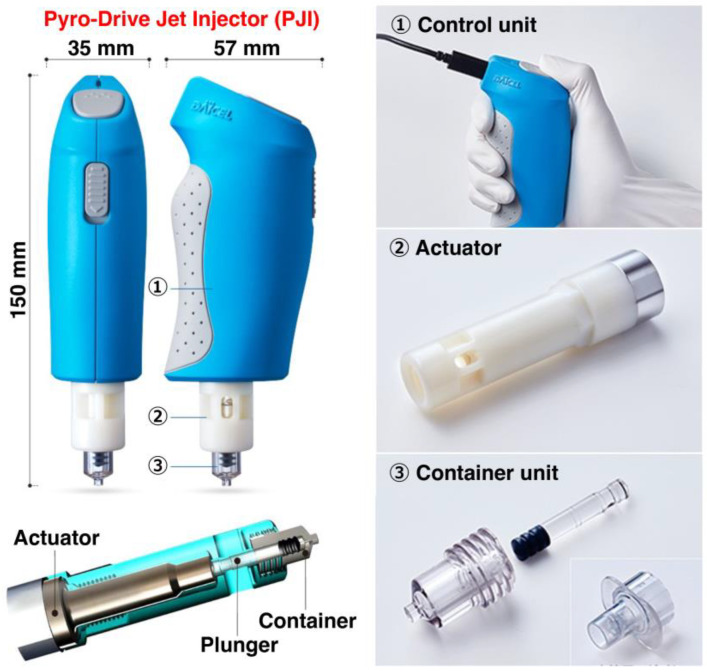
A diagram of the needle-free pyro-drive jet drive injector (PJI), called the Actranza™ lab (Daicel Corporation, Osaka, Japan, https://www.daicel.com/en/business/new-solution/actranza/, accessed on 1 April 2023). The PJI, which is composed of control unit, actuator, plunger and container, can successfully control two types of gunpowder with different times, weights and burning rates for the adjustment of the sample volume, the penetration depth and wide dispersion of delivery [42].

Very recently, a novel needle-free PJI called Actranza^TM^ lab (Daicel Corporation, Osaka, Japan) (Figure 3) has been developed, which has a unique characteristic in that the PJI utilizes gunpower as a mechanical driving force, and in particular bi-phasic pyrotechnics to induce high jet velocity and the wide dispersion of the injected plasmid DNA solution into the skin and muscle of mice, rats, pigs and humans [42,43,44,45,46,47,48,49,50,51]. The pyrotechnic technology was previously developed and initially utilized in the inflator products in an airbag [52] (https://www.daicel.com/safety/en/inflator/, accessed on 1 April 2023). An airbag is one of the safety restraint systems in cars, which rapidly fill with air to protect the driver from injuries when an accident occurs. The inflator is the most essential component in an airbag to generate gas and open the bag instantaneously through the combustion of propellant when the sensor receives a strong shock. The same pyrotechnic technology has been successfully and safely applied to develop the PJI [42]. Similar but another PJI called ZENEO^®^ (Crossject, Dijon, France), which is a needle-free auto-injector, has been previously developed [53]. The device was specifically designed for the self-administration of drugs and works via gas propulsion of the drug through micronozzles with 0.25–0.3 mm diameter. The actuator activates a nitrocellulose propellant-based gas generator, and the resulting gas propels the drug at high speed and pressure to penetrate the skin and deliver the drug intramuscularly. To date, whether this device is also useful and effective for vaccination remains to be addressed.

The Actranza^TM^ lab is composed of a control unit, actuator, plunger and container (Figure 3) and successfully controls two types of gunpowder with different times, weights and burning rates for the adjustment of sample volume, the penetration depth and the wide dispersion of the delivery [42,44,45,46]. The gas generator utilizes an ignition powder and a smokeless powder (Figure 2). Notably, by adjusting the weight ratio between the ignition powder and the smokeless powder, the delivery capacity of the PJI attains more than 95% of the ejected volume in the porcine skin, indicating almost no splash-back [42]. A single pyrotechnic driving force using one explosive or the other spring and gas methods cannot achieve such a radial dispersion of the injected solution [54].

In previous studies, when a fluorescence-labeled plasmid was intradermally injected into the rat or pig skin using the PJI, the plasmid DNA appeared to spread evenly from the epidermis to the dermis in the skin and highly colocalize with the nucleus [44,46]. In contrast, intradermal injection using a needled syringe spread the plasmid primarily only within the central area in the dermis, and it consequently colocalized much less with the nucleus. Thus, PJI more efficiently introduces the plasmid into the nucleus, resulting in higher protein expression than a needled syringe. Intriguingly, although the injection nozzle diameter is less than 0.2 mm, the plasmid DNA spreads radially over a 3 mm diameter. The high-speed camera analysis in a polyurethane gel revealed the possible mechanism (Figure 2B,C) [42]. The liquid rapid jet creates a hole in the gel upon the initial explosion caused by the ignition powder, penetrates and then ceases the penetration. Thereafter, upon the second explosion induced by the smokeless powder, the liquid jet disperses in a concentric circle around the end of the formed hole [42].

## 5. Acceleration of Antigen Uptake and Maturation by Shear Stress Generated with PJI-Mediated High Jet Velocity

A comparative analysis between the PJI and a needled syringe was performed with the luciferase activity detection assay. The same amount of luciferase expression vector plasmid was introduced into the skin intradermally. Results showed that approximately 10- and 40-fold higher luciferase expression was achieved by the PJI compared to a needled syringe in BALB/c mice and Caesarean Derived (CD) [Sprague Dawley (SD)] rats, respectively [44]. These enhanced protein expression levels are highly related to the injection speed. Rapid injection, with not only the jet injector but also even with a needled syringe, was reported to augment the shear stress and consequently increase the uptake of injected molecules in the cells by enhanced endocytosis in a receptor-mediated manner [55,56]. The flow rate induced by PJI was determined to be approximately 1 mL/s [42], which is much higher than that achieved by a needled syringe (less than 0.025 mL/s) [55]. The jet velocity after intradermal injection into the porcine skin is more than 200 m/s, which was similar to or higher than other injectors reported including those with spring, pressured gas, laser or electric driving force [41,42].

**Figure 4:**
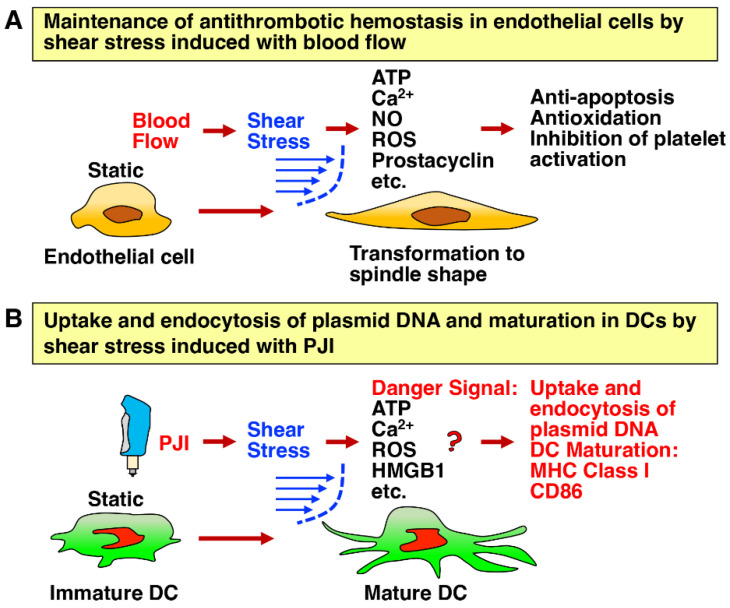
Shear stress generated by rapid jet velocity via the PJI is important for uptake and endocytosis of plasmid DNA and presumably DC maturation. (**A**) Blood flow generates shear stress applied tangentially or parallel to the surface of endothelial cells [57]. Endothelial cells are critical sensors in response to shear stress, which physiologically maintains their antithrombotic homeostasis. (**B**) Similarly, shear stress generated by rapid jet velocity of the PJI accelerates the uptake and endocytosis of plasmid DNA, resulting in augmented protein expression in DCs [43,58,59]. Moreover, shear stress presumably plays an important role in the activation of innate immunity, including the induction of DC maturation, leading to the establishment of adaptive immunity [61,62,63].

Blood flow is well known to create a frictional force applied tangentially or parallel to the surface of cells, which is called shear stress (Figure 4A) [57]. Endothelial cells are critical sensors in the response to shear stress, which physiologically regulates the homeostasis of thrombosis and fibrosis; higher shear stress is protective from thrombosis, and lower shear stress promotes thrombosis and consequently leads to atherosclerosis [57]. In addition, shear stress has been reported to facilitate endocytosis and the uptake of extracellular molecules, including DNA (Figure 4B) [58,59]. When the luciferase-expression plasmid was first intradermally injected with a needled syringe, the subsequent injection of saline into the same place by PJI greatly enhanced the luciferase activity compared to that by a needled syringe [43]. This effect is highly likely to be due to the increased uptake of the plasmid by the shear stress generated by PJI. Supporting this, the induction of luciferase activity was inhibited by heparin, which is an inhibitor of the uptake of injected DNA [55], and also suppressed by several typical inhibitors of endocytosis [43]. Conversely, the treatment with chloroquine, which induces endosome escape by rupturing endosomal vesicles via the protonation of endosomes in an acidic environment [60], increased the induction of luciferase activity.

The mechanism whereby the shear stress induces the uptake of the injected DNA is presumably considered as follows. Shear stress elicits torsion in the hydrophilic lipid of the cellular membrane and instability of the membrane bilayer and, consequently, induces the formation of transient pores in the membrane through which the injected DNA solution is taken up [64,65,66]. Interestingly, such techniques of shear stress-induced deformation of cells have been successfully applied to the new system of microfluidic and nanofluidic intracellular delivery of plasmid DNA, RNA and proteins into the cytosol or nucleus of the cells [65,66]. Thus, shear stress induced by the rapid jet velocity of the PJI enhances the uptake and endocytosis of plasmid DNA, leading to augmented protein expression. Of note, shear stress was also reported to induce translocation of HMGB1 [61], ATP release [62] and reactive oxygen species (ROS) generation [63] in endothelial cells, which has prompted us to conceive of the activation of innate immunity by shear stress as discussed later.

## 6. PJI-Mediated Vaccination against Cancers with Augmented Cellular Immunity

Cancer vaccination is more complicated than vaccination against pathogens. This is because pathogens, such as viruses or bacteria, are foreign to the human immune system, while cancers are derived from normal and healthy cells; therefore, it is difficult to distinguish between normal cells and cancer cells. Vaccines can induce prophylactic effects that prevent the future infection as well as induce therapeutic effects against existing cancers.

In a cancer experimental model, ovalbumin (OVA) was commonly used as an antigen, and the inoculation of C57BL/6 mice with tumor cells that were stably transfected with the OVA-expression vectors (lymphoma E.G7-OVA or melanoma B16F10-OVA) induced OVA-specific cellular and humoral immune responses. In a comparative study between the PJI and a needled syringe, the effectiveness of the induction of OVA-specific cellular and humoral immune responses by intradermal injection of OVA-expression plasmid (pOVA obtained from Addgene) was examined [50]. After a single intradermal injection of OVA-expression plasmid, OVA-specific cytotoxic CD8^+^ T cells (CTLs) were rapidly generated within 2 weeks, which was detected by a tetramer assay using flow cytometry. OVA-specific CD4^+^ T cells appeared to be more slowly activated, differentiating into both Th1 and Th2 cells. Consistent with the data, OVA-specific in vivo killing activity was induced, followed by OVA-specific antibody production, including both IgG2a and IgG1 isotypes [50]. The induction of the OVA-specific cellular immunity by the PJI was much higher than that by a needled syringe [50]. This is highly likely to be explained by the fact that the PJI induces a much higher shear stress and consequently increased expression of the target protein OVA in the dermal DCs due to a higher jet velocity and a wider dispersion of injected solution containing the OVA-expression plasmid [50].

Moreover, two intradermal injections of the OVA-expression plasmid into C57BL/6 mice at a 2-week interval greatly induced prophylactic effects against inoculation with E.G7-OVA tumor cells [50]. Mice that were first inoculated with the tumor and then 3 days later intradermally injected with the OVA-expression plasmid pOVA exhibited retarded tumor growth, suggesting therapeutic effects. Moreover, mice injected intradermally with OVA-expression plasmid only once exhibited strong prophylactic effects on the growth of the tumor inoculated one week later [50]. Interestingly, this prophylactic effect was stronger than that found for injection with OVA protein precipitated with aluminum hydroxide (alum/OVA) or OVA protein emulsified with complete Freund’s adjuvant (CFA/OVA). This prophylactic effect was highly dependent on CD8^+^ T cells and generation of CTL because the depletion of CD8^+^ T cells using their specific antibody almost canceled the prophylactic effect [50]. This effect could be attributed to the potent induction of OVA-specific CD8^+^ T cells by the PJI although the kinetics of the induction by the PJI were much higher than those by CFA/OVA but slightly lower than those by alum/OVA [50]. However, the induction level by the PJI was higher than that by alum/OVA or CFA/OVA.

**Figure 5:**
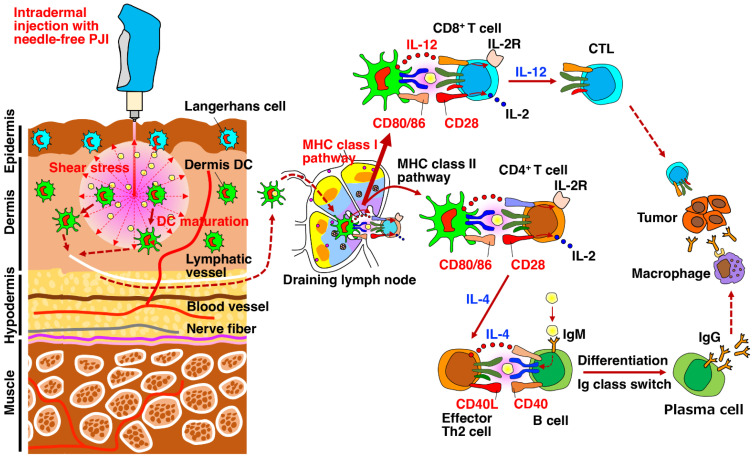
Possible molecular mechanism whereby the intradermal injection with PJI induces potent antigen-specific cellular and humoral immunity. Intradermal injection of OVA-expression plasmid DNA using the PJI elicits shear stress via its high jet velocity and resultant wide dispersion of the DNA solution in the skin [43]. Then the shear stress accelerates the uptake and endocytosis of plasmid DNA into the nucleus of the skin cells, including DCs. Consequently, the shear stress increases the target OVA protein expression and presentation of OVA protein-processed peptides through the MHC class I pathway in DCs. Simultaneously, the shear stress presumably provokes danger signals [61,62,63], which together with adjuvant activity of the plasmid DNA induce the maturation of DCs with upregulation of CD86 [67] and CCR7 [68], followed by migration into the draining lymph nodes. Then mature DCs presenting OVA peptides on the MHC class I stimulate naive CD8^+^ T cells and consequently generate effector CTLs [69]. On the other hand, the phagocytosis of dead cells expressing OVA or OVA protein by immature DCs activates the MHC class II pathway, subsequently upregulates CD86 and CCR7 on the DCs and subsequently induces their migration into the draining lymph nodes [69]. There, mature DCs presenting OVA peptides on the MHC class II stimulate naive CD4^+^ T cells and consequently generate effector CD4^+^ T cells, such as Th2 cells, resulting in the stimulation of OVA-specific B cells and differentiation into antibody-producing plasma cells.

Collectively, the possible molecular mechanism whereby the PJI augments the antitumor effects of vaccination with OVA-expression plasmid is presumably considered as follows (Figure 5). The intradermal injection of OVA-expression plasmid using the PJI generates shear stress via its high jet velocity, which induces the wide dispersion of the DNA solution in the skin (Figure 2C) [43]. Then the shear stress accelerates the uptake and endocytosis of plasmid DNA into the nucleus of the skin cells, including DCs. Consequently, the shear stress increases the target OVA protein expression and presentation of the OVA protein-processed peptides via the MHC class I pathway in DCs. Simultaneously, the shear stress presumably provokes danger signals such as HMGB1 [61], ATP [62] and ROS [63], which together with adjuvant activity of the plasmid DNA induce the maturation of DCs with upregulation of CD86 [67] and CCR7 [68], followed by the migration into the draining lymph nodes. Then mature DCs presenting OVA peptides on the MHC class I stimulate naive CD8^+^ T cells and consequently generate effector CTLs [69]. On the other hand, the phagocytosis of dead cells expressing OVA or OVA protein by immature DCs activates the MHC class II pathway, subsequently upregulates CD86 and CCR7 on the DCs and induces their migration into the draining lymph nodes [69]. There, mature DCs presenting OVA peptides on the MHC class II stimulate naive CD4^+^ T cells and consequently generate effector CD4^+^ T cells, such as Th2 cells, resulting in the stimulation of OVA-specific B cells and differentiation into antibody-producing plasma cells. Thus, the intradermal injection of OVA-expression plasmid using the PJI efficiently augments both antitumor cellular and humoral immunity in an antigen-specific manner.

## 7. PJI-Mediated Vaccination against Pathogen SARS-COV-2 with Enhanced Humoral Immunity

The development of a safe and effective vaccine against SARS-CoV-2 and its mutants is urgent due to the global prevalence of the COVID-19 pandemic. Several vaccine candidates have been developed, including mRNA, DNA and protein subunits [3]. Recent advancements in the generic manufacturing techniques have accelerated the rapid production of mRNA and DNA vaccines against the SARS-CoV-2 S protein, which can adapt to viral mutations [3,25,70]. Due to the fact that DNA vaccines are thermo-stable and do not need a cold chain logistics system and deep-freeze storage that are required for mRNA vaccines, DNA vaccine against SARS-CoV-2 is also a good alternative candidate [71]. Multiple intradermal injections of the OVA-expression plasmid pOVA using the PJI induced the dose-dependent production of antibodies against OVA, which was more than a hundred times higher than that using a needled syringe without alum adjuvant in CD (SD) rats and BALB/c mice [44,50]. In a preclinical study, intramuscular injection of the plasmid DNA vaccine (pVAX1-SARS-CoV2-co, AG0302-COVID19, Daicel and AnGes, Osaka, Japan) against SARS-CoV-2 S protein with an alum adjuvant using a needled syringe displayed enhanced humoral and cellular immune responses [72]. Similarly, the intradermal injection of the same plasmid into C57BL/6 mice and CD (SD) rats using the PJI without any adjuvants increased the antibody production against S protein, and the generation of S protein-specific IFN-γ producing cells [47]. In addition, the intradermal injection augmented the inhibitory effect of the serum harvested from the injected mice on the binding of human ACE2 to the S1 and S2 protein and showed the protection from COVID-19 infection without any serious disorders [47].

Given the rapid emergence of new SARS-CoV-2 variants of concern such as the delta and omicron variants, the prompt production of modified vaccines adapted to them is urgent. The modified DNA vaccine (GPΔ-DNA vaccine, Daicel and AnGes, Osaka, Japan) targeting the delta variant and carrying S protein mutations based on the delta variant was constructed. The efficacy of intradermal injection using the PJI without any adjuvants was compared to that of intramuscular injection with an alum adjuvant using a needled syringe in CD (SD) rats [48]. The intradermal injection using the PJI without any adjuvants induced significantly higher antibody titer and neutralizing activity against vesicular stomatitis virus (VSV)-based pseudo-typed viruses carrying the SARS-CoV-2 S protein delta variant than that of the intramuscular injection even with an alum adjuvant using a needled syringe [48]. Moreover, the intradermal injection using PJI protected human ACE2 knock-in mice from delta variant infection. Thus, DNA vaccines can be rapidly adapted to newly emerging variants, and the PJI can further improve their efficacy.

A phase I study was then conducted to assess the safety and immunogenicity of intradermally injected DNA vaccine (AG0302-COVID-19) with two doses using the PJI twice at a 2-week interval [49]. No evident safety problems were observed, and the cellular immune responses, such as IFN-γ production of peripheral blood mononuclear cells in response to the S protein, were observed in some subjects. Nevertheless, under these clinical trial conditions, antibody production was barely enhanced, possibly due to the difference in species and insufficient antigen expression caused by the injection of these low doses [49].

## 8. Potential Applications of PJI in Other Research Fields

**Figure 6:**
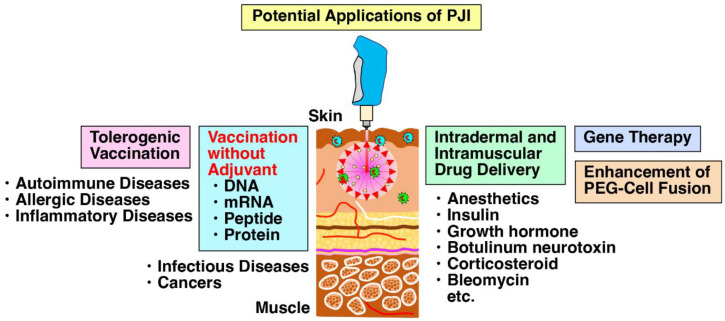
PJI is a versatile device having a variety of possible applications in many research fields. In addition to vaccination of plasmid DNA, mRNA, peptide and protein without any adjuvants, the PJI can be applied to a variety of research fields as a tool for efficient delivery, such as tolerogenic vaccination [74], intradermal and intramuscular delivery of drugs [39,40,73], gene therapy [75,76] and enhancement of PEG-cell fusion [51].

The PJI is a superior device as a vaccinating tool to augment immunity against cancers and pathogens and also has a variety of potential applications in many research fields (Figure 6). Very recently, the PJI has been reported to be successfully applied to gene therapy by intramuscular delivery of plasmid DNA of basic fibroblast growth factor (bFGF) into muscle tissues of the ischemia-induced muscle injury model mice [45]. Intramuscular delivery of the plasmid DNA directly into the hearts of normal mice using the PJI also augmented its expression and the angiogenesis [45]. Whether or not the intramuscular injection induces shear stress remains to be examined. Moreover, the application of transient pressure induced by the PJI has significantly improved polyethylene glycol (PEG)-cell fusion efficiency compared to the conventional PEG method, resulting in enhanced DC-tumor cell fusion vaccine in mice and higher generation of hybridoma cell preparation [51]. Further possible application examples are as follows: intradermal delivery of drugs, such as anesthetics, insulin, growth factors, botulinum neurotoxin [73] and bleomycin [39,40]; tolerogenic vaccination by adding immunosuppressive compounds, such as dexamethasone and rapamycin [74]; coexpression of immune suppressive cytokines, such as IL-10, IL-27 or IL-35 to induce IL-10-producing regulatory T and B cells and IL-35-producing regulatory T and B cells [75,76]; and vaccination of mRNA, peptide and protein, in addition to DNA.

Due to the fact that the injection of intact mRNA induces strong inflammatory responses, mRNA, whose uridine nucleotide is replaced with pseudouridine to reduce the inflammatory responses, has been synthesized and this replacement concomitantly decreases its antigenicity as well [77]. Therefore, to reverse the decreased antigenicity and delivery into cells efficiently, the modified mRNA needs to be encapsulated in LNPs [10]. However, adverse effects caused by COVID-19 vaccination are suggested to mainly stem from the highly inflammatory nature of the ionizable lipid component of the LNPs although it is necessary for its adjuvanticity [11]. Therefore, the PJI may be one of the alternative candidates to LNPs to augment not only the delivery of the modified mRNA into cells but also its antigenicity. Finally, to prove that the PJI is possibly one of the physical adjuvants, the intradermal injection of proteins using the PJI is interesting. As expected, our preliminary data suggest that the intradermal injection of endotoxin-free OVA protein using the PJI without supplying any additional adjuvants augmented the generation of OVA-specific CTLs (Mizoguchi et al. manuscript in preparation). This implies that the development of safer and more efficient adjuvant-free vaccination of protein antigens with the needle-free PJI could be highly feasible.

## 9. Conclusions

The novel needle-free PJI is a promising efficient and safe tool as a possibly powerful physical adjuvant for vaccination with DNA, mRNA and proteins. The PJI induces potent protective cellular and humoral immunity against cancers and pathogens without any chemical and biological adjuvants. Moreover, this PJI is a versatile delivery tool having a variety of potential applications in many research fields although the cost and expertise requirements of using the PJI might need to be further considered.

## Data Availability

Not applicable.
